# Retraction: MicroRNA-214 Suppresses Oncogenesis and Exerts Impact on Prognosis by Targeting PDRG1 in Bladder Cancer

**DOI:** 10.1371/journal.pone.0244274

**Published:** 2020-12-17

**Authors:** The PLOS ONE Editors

Following the publication of this article [1], concerns were raised regarding the results presented in Figs 3 and 6.

Specifically,

- The 5637 miR-NC-0h panel of Fig 3A partially overlaps with the 5637 miR-214-0h panel of Fig 3A.
- The 5637 miR-214-0h panel of Fig 3A partially overlaps with the 5637 si-NC-0h panel of Fig 6A.
- The T24 si-NC-0h panel of Fig 6A partially overlaps with the T24 si-PDRG1-0h panel of Fig 6A.
- Partial overlap has been detected between the T24-miR-214 panel of Fig 3B, the T24-miR-214 panel of Fig 3C, the T24-si-PDRG1 panel of Fig 6B and the T24-si-PDRG1 panel of Fig 6C.

The authors stated that the similarity observed between the panels described above are the result of inadvertent errors introduced during figure preparation and provided data underlying their published results. However, the extent of the image concerns observed in this article raise serious concerns regarding the handling of the data obtained during this study and the integrity of the published results. In light of the concerns affecting multiple figure panels that question the integrity of these data, the *PLOS ONE* Editors retract this article.

XZ agreed with the retraction. LW, YY, ZD, HW, and LD either did not respond directly or could not be reached. JW and CW did not agree with the retraction.
